# Complete genome sequence of *Pseudomonas brassicacearum* strain L13-6-12, a biological control agent from the rhizosphere of potato

**DOI:** 10.1186/s40793-016-0215-1

**Published:** 2017-01-09

**Authors:** Christin Zachow, Henry Müller, Jana Monk, Gabriele Berg

**Affiliations:** 1 Austrian Centre of Industrial Biotechnology (ACIB GmbH), Petersgasse 14, 8010 Graz, Austria; 2 Institute of Environmental Biotechnology, Graz University of Technology, Petersgasse 12, 8010 Graz, Austria; 3 Faculty of Agriculture and Life Sciences, Department of Ecology, Lincoln University, Ellesmere Junction Road, Lincoln, 7647 New Zealand

**Keywords:** Short genome report, *Pseudomonadaceae*, *Pseudomonas brassicacearum* L13-6-12, Potato rhizosphere, Volatile organic compounds, Biocontrol, Plant growth promotion, Secretion systems

## Abstract

*Pseudomonas brassicacearum* strain L13-6-12 is a rhizosphere colonizer of potato, lettuce and sugar beet. Previous studies have shown that this motile, Gram-negative, non-sporulating bacterium is an effective biocontrol agent against different phytopathogens. Here, we announce and describe the complete genome sequence of *P. brassicacearum* L13-6-12 consisting of a single 6.7 Mb circular chromosome that consists of 5773 protein coding genes and 85 RNA-only encoding genes. Genome analysis revealed genes encoding specialized functions for pathogen suppression, thriving in the rhizosphere and interacting with eukaryotic organisms.

## Introduction

*Pseudomonas brassicacearum* strain L13-6-12 was isolated from the rhizosphere of a field grown potato plant [1]. L13-6-12 was selected as effective biological control agent with disease-suppressing effects against *Rhizoctonia solani* Kühn in treated lettuce and potato plants in greenhouse and field trials [2]. It has additional antifungal activity against the phytopathogenic fungi *Alternaria alternata* *,* *Botrytis cinerea* Pers. DSM5145 *,* *Penicillium italicum* *,* *Phoma betae* *,* *Sclerotinia sclerotiorum* *,* *Verticillium dahliae* Kleb. V25 (all Ascomycota) and *Rhizoctonia solani* AG2-2IIIB and AG4 and *Sclerotium rolfsii* (Basidiomycota). This biocontrol activity is linked to the production of secondary metabolites, including 2,4-diacetylphloroglucinol and hydrogen cyanide. For various strains of plant-associated pseudomonads the production of antifungal metabolites like DAPG and recombinase genes were identified as the major trait for biological control of soilborne pathogens and plant root colonization [3]. Genes in L13-6-12 predicting functions for biocontrol include factors such as secreted proteases and comprehensive secretion systems. It also supports plant growth by nutrient delivery by phosphate solubilization, production of indole-3-acetic acid as well as by aminocyclopropane-1-carboxylate deaminase activity. Additionally, L13-6-12 copes with abiotic stresses such as desiccation and high salt concentrations. To gain insight into ecological relevant traits and to improve its biotechnological applications we sequenced the complete genome of this bacterium.

## Organism information

### Classification and features

**Fig. 1:**
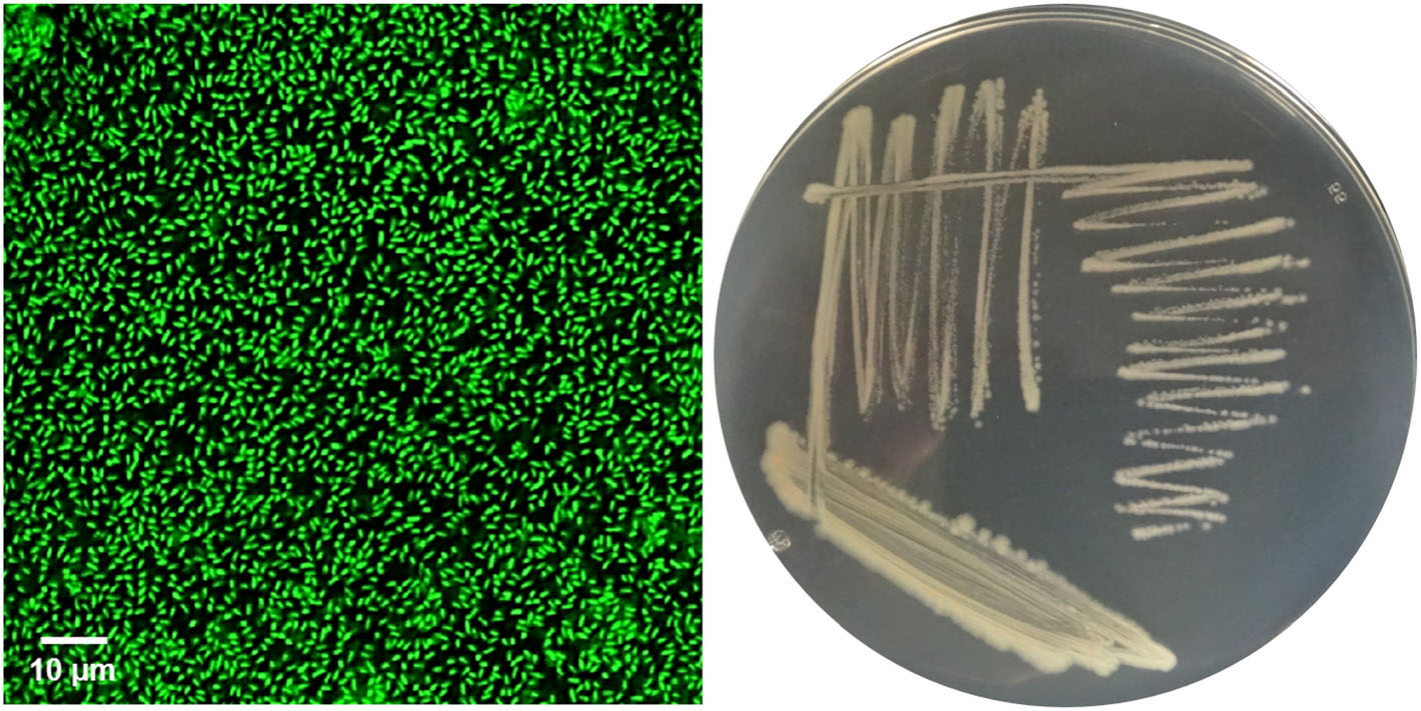
Photomicrographs of source organism. Images of *P. brassicacearum* L13-6-12 cells using confocal laser scanning microscopy (CLSM, *left*) and the appearance of colony morphology after 48 h growing on NB agar at 25 °C (*right*). Image was obtained using acridin orange (0.4 mg ml^−1^ water) stained L13-6-12 cells with 40× magnification. Cells were visualized with Leica TCS SP CLSM (Leica Microsystems, Wetzlar, Germany) and analysed using Leica Application Suite Advanced Fluorescence (LAS AF) software Version 3.5

*P. brassicacearum* L13-6-12 is a motile, Gram-negative, non-sporulating rod in the order *Pseudomonadales* of the class *Gammaproteobacteria*. The rod-shaped cells are approximately 0.4 μm in width and 0.8–1.5 μm in length (Fig. 1 left). The strain is moderately fast-growing, forming 1 mm colonies within 1–2 days at 25 °C. Colonies formed on NBII agar plates are yellow shining, domed and moderately mucoid with smooth margins (Fig. 1 right). Cultivation for more than two weeks on NA result in a color change of the medium to dark brown. L13-6-12 was isolated from a potato rhizosphere from plants grown in a field trial in Groß Lüsewitz, Germany, in 1997 [1].

Even though the optimal growth temperature is 30 °C, L13-6-12 can also slowly replicate at 5 °C in liquid Luria Bertani medium. Growth was observed at 37 °C and slightly at 40 °C in this culturing medium as well as on solidified medium after 24 h. The strain grows in complex media, but not in Standard Succinate Medium (pH 7.0). Optimum pH for growth in LB is pH 7.0. The bacterium is an efficient colonizer of lettuce, potato [2, 3] and sugar beet plants, where microcolonies consisted of tens to hundreds of bacterial cells, forming an interconnected network between epidermal cells in the rhizoplane [3]. It does not cause any deleterious effect on its original host plant potato or lettuce [1, 2] and sugar beet [4] or on the nematode *Caenorhabditis elegans* [5]. Strain L13-6-12 has natural resistance to gentamycin (10 μg mL^−1^), trimethoprim (50 μg mL^−1^) and is able to develop spontaneous rifampicin-resistance.

**Fig. 2:**
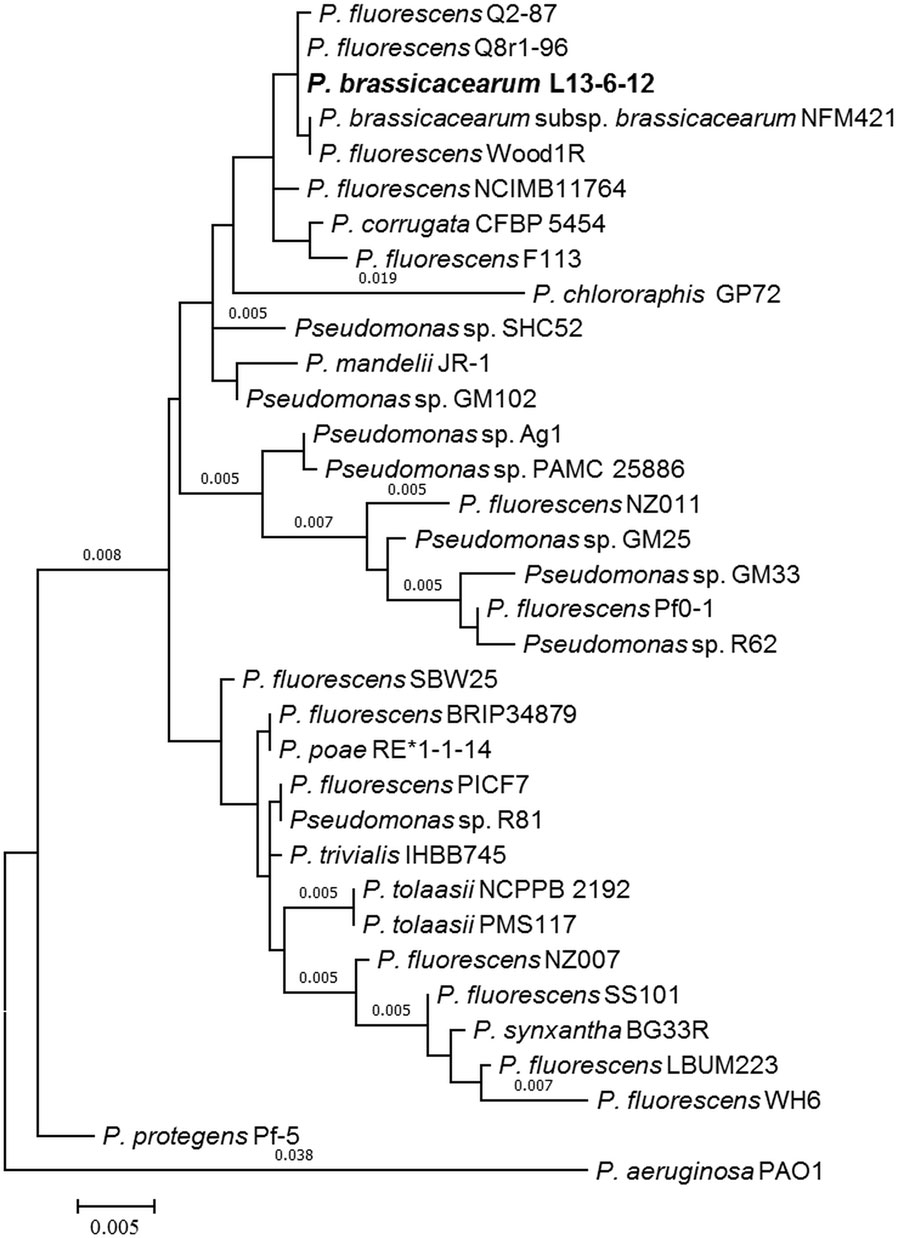
Phylogenetic tree showing the position of *P. brassicacearum* L13-6-12 in relationships among other strains of *Pseudomonas* spp. including *P. aeruginosa* PAO1 as outgroup. The tree is based on 16S rRNA gene alignments and was conducted in MEGA6 [41]. Initial tree for the heuristic search were obtained automatically by applying Neighbor-Join and BioNJ algorithms to a matrix of pairwise distances estimated using the Maximum Composite Likelihood approach, and then selecting the topology with superior log likelihood value

Minimum Information about the Genome Sequence of *P. brassicacearum* L13-6-12 is summarized in Table 1. The phylogenetic relationship of *P. brassicacearum* L13-6-12 to other species within the genus *Pseudomonas* is visualized in a 16S rRNA based tree (Fig. 2) [6].

**Table 1:** Classification and general features of *P. brassicacearum* strain L13-6-12 according to the MIGS recommendation [29]

MIGS ID	Property	Term	Evidence code^a^
	Classification	Domain *Bacteria*	TAS [30]
		Phylum *Proteobacteria*	TAS [31]
		Class *Gammaproteobacteria*	TAS [32]
		Order *Pseudomonadales*	TAS [33, 34]
		Family *Pseudomonadaceae*	TAS [31, 35]
		Genus *Pseudomonas*	TAS [36–39]
		Species *Pseudomonas brassicacearum*	TAS [39]
		Strain: L13-6-12	TAS [1]
	Gram stain	Negative	IDA, TAS [39]
	Cell shape	Rod	IDA, TAS [39]
	Motility	Motile	TAS [39]
	Sporulation	Not reported	NAS
	Temperature range	5 °C–40 °C	IDA
	Optimum temperature	30 °C	IDA
	pH range; Optimum	5.0–9.0; 7	IDA
	Carbon source	Heterotrophic	TAS [39]
MIGS-6	Habitat	Potato, Rhizosphere	TAS [1]
MIGS-6.3	Salinity	1.0–9.0% NaCl (w/v)	IDA, TAS [1]
MIGS-22	Oxygen requirement	Aerobic	TAS [39]
MIGS-15	Biotic relationship	Rhizospheric	TAS [1, 2, 4]
MIGS-14	Pathogenicity	Non-pathogen	TAS [1, 5]
MIGS-4	Geographic location	Gross Luesewitz, Germany	TAS [1]
MIGS-5	Sample collection	2001	TAS [1]
MIGS-4.1	Latitude	54°4′15.4704” N	NAS
MIGS-4.2	Longitude	12°20′19.9248” E	NAS
MIGS-4.4	Altitude	37 m	NAS

^a^Evidence codes - IDA: Inferred from Direct Assay; TAS: Traceable Author Statement (i.e., a direct report exists in the literature); NAS: Non-traceable Author Statement (i.e., not directly observed for the living, isolated sample, but based on a generally accepted property for the species, or anecdotal evidence). These evidence codes are from the Gene Ontology project [40]

## Genome sequencing information

### Genome project history

Strain L13-6-12 was originally assigned to *P. fluorescens* based on 16S rRNA gene sequencing and alignments with NCBI database [1, 2, 4, 5]. After average nucleotide identity [7] comparison of the genome sequence against the genomes of the type strains and proxytype strains that are already in GenBank, L13-6-12 showed 99.604% identity to the type genome of *P. brassicacearum* with 95.5% coverage of the genome. The genome of *P. brassicacearum* strain L13-6-12 was selected for sequencing based on its ability to exert biocontrol abilities against fungal pathogens and to promote plant growth [1, 3]. This whole-genome shotgun project has been deposited in the NCBI database under the accession no. CP014693. The version described in this paper is the first version (Table 2).

**Table 2:** Project information

MIGS ID	Property	Term
MIGS 31	Finishing quality	Finished
MIGS-28	Libraries used	PacBio RS libraries with inserts of 8 to 20 kb
MIGS 29	Sequencing platforms	PacBio RS II sequencer
MIGS 31.2	Fold coverage	84.9
MIGS 30	Assemblers	Hierarchical Genome Assembly Process algorithm implemented in the PacBio SMRT Analysis software
MIGS 32	Gene calling method	Glimmer gene prediction, NCBI Prokaryotic Genome Annotation Pipeline
	Locus Tag	A0U95
	Genbank ID	CP014693
	GenBank Date of Release	September 20, 2016
	GOLD ID	Gs0118536, Gp0137088
	BIOPROJECT	PRJNA311625
MIGS 13	Source Material Identifier	L13-6-12
	Project relevance	Plant-bacteria interaction, agricultural, environmental

### Growth conditions and genomic DNA preparation

*P. brassicacearum* strain L13-6-12 was grown in 50 mL of NBII (Sifin, Berlin, Germany) medium and incubated for 20 h at 30 °C. 1.0 mL was centrifuged at 2500 × g for 5 min at 4 °C and genomic DNA was extracted using the MasterPure DNA purification kit (Epicentre, Madison, WI, USA). DNA quality and quantity were validated by agarose gel electrophoresis and spectrophotometry using a UV-Vis spectrophotometer (NanoDrop 2000c, Thermo Fisher Scientific, Waltham, MA USA). In total, 54 μg genomic DNA (1.8 μg μL^−1^) was sent on dry ice to the sequencing service. PacBio RS libraries with inserts of 8 to 20 kb were constructed and sequenced at GATC Biotech (Konstanz, Germany).

### Genome sequencing and assembly

PacBio RS libraries with inserts of 8 to 20 kb were constructed and sequenced at GATC Biotech (Konstanz, Germany) using single molecule, real-time sequencing. Assembly was completed with the Hierarchical Genome Assembly Process algorithm implemented in the PacBio SMRT Analysis software (Pacific Biosciences, Menlo Park, CA, USA). The assembly of L13-6-12 genome based on 130,283 quality reads with a mean length of 4995 bp resulting in a single circular chromosome consisting of 6,715,909 bp, with 84.9-fold overall coverage and a GC content of 60.7%.

### Genome annotation

Automatic annotation was conducted on the RAST Web server (version 36) using RAST gene calling based on FIGfam version Release70 [8, 9], and additional annotation for using the automated assignment of COG-functions to protein-coding genes was completed on the BASys web server using Glimmer gene prediction [10, 11]. Pseudogenes were predicted using the NCBI Prokaryotic Genome Annotation Pipeline. Signal peptides and transmembrane helices were predicted using SignalP [12, 13] and TMHMM [14, 15].

## Genome properties

**Fig. 3:**
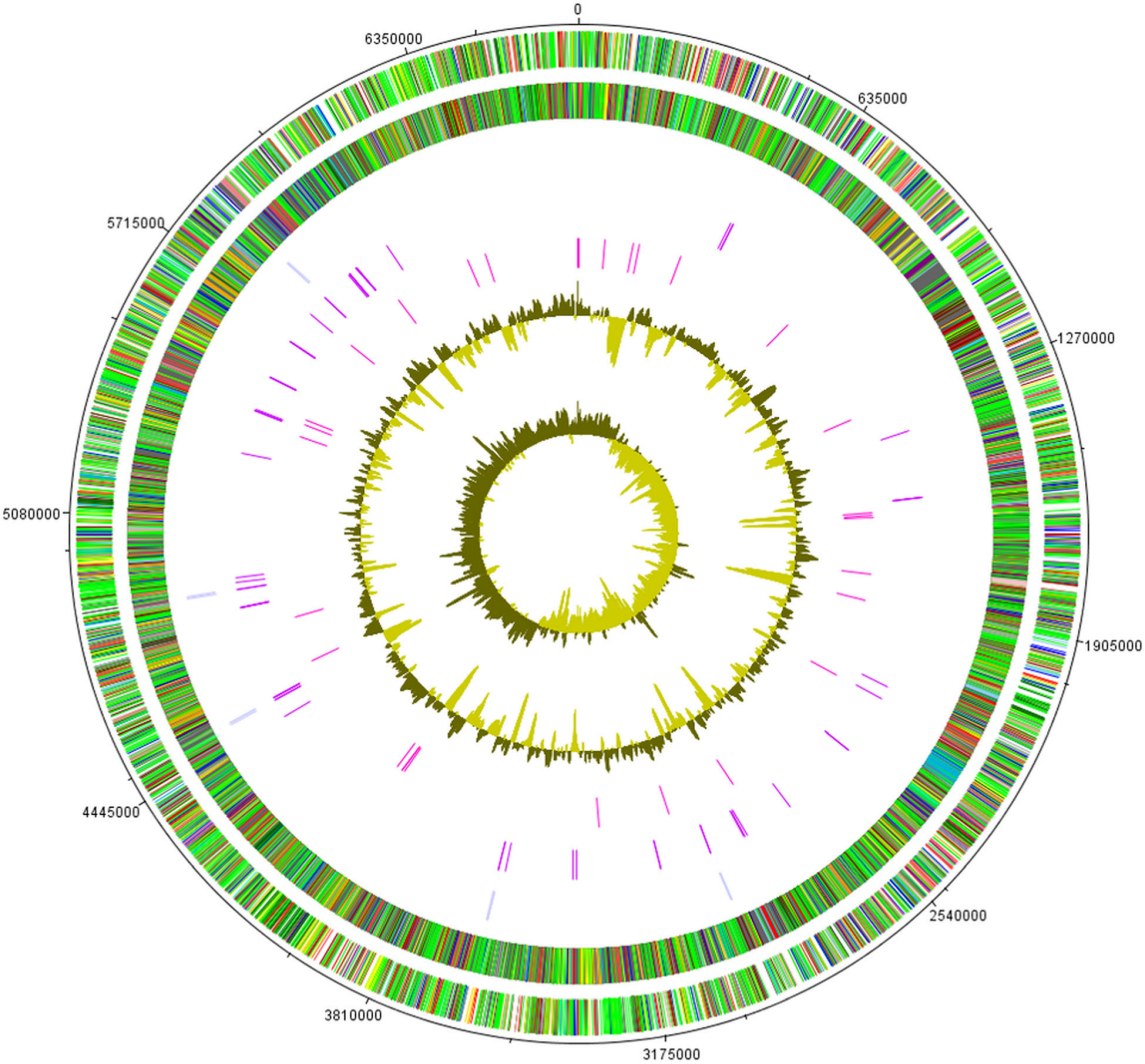
Graphical map of the chromosome. The outer scale is marked every 50 kb. Circles range from 1 (*outer circle*) to 7 (*inner circle*). Circle 1 and 2, ORFs encoded by leading and lagging strand respectively, with color code for functions: salmon, translation, ribosomal structure and biogenesis; aquamarine, RNA processing and modification; light blue, transcription; cyan, DNA replication, recombination and repair; tan, chromatin structure and dynamics; turquoise, cell division; dark orange, defense mechanisms; deep pink, post-translational modification, protein turnover and chaperones; dark olive green, cell envelope biogenesis; purple, cell motility and secretion; lavender, intracellular trafficking, secretion, and vesicular transport; forest green, inorganic ion transport and metabolism; pink, signal transduction; red, energy production; sienna, carbohydrate transport and metabolism; yellow, amino acid transport; orange, nucleotide transport and metabolism; gold, co-enzyme transport and metabolism; cornflower blue, lipid metabolism; blue, secondary metabolites, transport and catabolism; gray, general function prediction only; yellow green, unknown function; black, function unclassified or unknown. Circle 3 and 4, distributions of tRNA genes and rrn operons respectively. Circle 5, distribution of pseudogenes. Circle 6 and 7, G + C content and GC skew (G-C/G + C) respectively

The genome of L13-6-12 is composed of one circular chromosome consisting of 6,715,909 bp with an average GC content of 60.7% (Table 3 and Fig. 3), which is similar to that of other *P. brassicacearum* strains. Among the 5887 predicted genes, 5773 were identified as protein coding genes. Of the last, 4801 (83.2%) were assigned a putative function, while the other 972 (16.8%) were designated as hypothetical proteins. The classification of CDSs into functional categories according to the COG [16, 17] database is summarized in Table 4 based on BASys gene prediction. Beside the predicted genes, the genome annotation contained 65 tRNA, five rRNA loci (5S, 16S, 23S) with one additional 5S rRNA, four ncRNAs and 284 predicted SEED subsystem features.

**Table 3:** Genome statistics

Attribute	Value	% of Total
Genome size (bp)	6,715,909	100
DNA coding (bp)	6,050,433	90.1
DNA G + C (bp)	4,091,158	60.7
DNA scaffolds	1	–
Total genes	5887	100
Protein coding genes	5773	98.1
RNA genes	85	1.4
Pseudo genes	29	0.5
Genes in internal clusters	NA	–
Genes with function prediction	4801	83.2
Genes assigned to COGs	4481	77.6
Genes with Pfam domains	3770	65.3
Genes with signal peptides	390	6.8
Genes with transmembrane helices	1389	24.1
CRISPR repeats	NA	–

**Table 4:** Number of genes associated with general COG functional categories

Code	Value	%age	Description
J	2	0.03	Translation, ribosomal structure and biogenesis
A	3	0.04	RNA processing and modification
K	281	4.21	Transcription
L	32	0.48	Replication, recombination and repair
B	545	8.16	Chromatin structure and dynamics
D	81	1.21	Cell cycle control, Cell division, chromosome partitioning
V	284	4.25	Defense mechanisms
T	162	2.43	Signal transduction mechanisms
M	211	3.16	Cell wall/membrane biogenesis
N	165	2.47	Cell motility
U	442	6.62	Intracellular trafficking and secretion
O	153	2.29	Posttranslational modification, protein turnover, chaperones
C	256	3.83	Energy production and conversion
G	158	2.37	Carbohydrate transport and metabolism
E	174	2.61	Amino acid transport and metabolism
F	239	3.58	Nucleotide transport and metabolism
H	112	1.68	Coenzyme transport and metabolism
I	468	7.01	Lipid transport and metabolism
P	344	5.15	Inorganic ion transport and metabolism
Q	263	3.94	Secondary metabolites biosynthesis, transport and catabolism
R	50	0.75	General function prediction only
S	56	0.84	Function unknown
–	3201	47.93	Not in COGs

The total is based on the total number of protein coding genes in the genome based on BASys gene prediction

## Insights from the genome sequence

The genome-wide phylogenetic analysis on different *Pseudomonas* species with the L13-6-12 genome showed that strain L13-6-12 clusters closely to *P. fluorescens* Q8r1-96 (NCBI Accession no. PRJNA67537) (Fig. 2). Recently, Q8r1-96 was described as a biological control strain that produces the antibiotic DAPG and that exceptionally colonizes the roots of wheat and pea [18, 19]. The genome of L13-6-12 contains several genes, which are important contributors to biological control. They are related to the biosynthesis of secondary metabolites or antimicrobial products that are similar to those found in the genomes of other Pseudomonads [20]. We detected genes for the biosynthesis of DAPG (Locus tags: A0U95_04640, A0U95_04655, A0U95_04660, A0U95_04665) and productions of exoproteases (A0U95_00125, A0U95_02755). The suppression of hyphal growth of *S. rolfsii* by volatile organic compounds produced by L13-6-12 was observed in a test system developed by Cernava et al. [21]. Volatile components have been shown to act as antibiotics and to induce plant growth [22, 23]. Hydrogen cyanide (HCN) is an inorganic volatile compound with antagonistic effects against soil microbes [24]. The production of HCN was observed in L13-6-12 (A0U95_28525) by applying an assay according to Blom et al. [25]. Genes predicting biosynthesis of other volatile components such as 2,3-butanediol (A0U95_29290) and acetoin (A0U95_29285) were found as well.

We further identified genes most probably involved in the direct promotion of plant growth, such as biosynthesis or carrier gene clusters for spermidine (A0U95_07830), pyoverdine (e.g. A0U95_07605, A0U95_25745, A0U95_25750) and aminocyclopropane-1-carboxylate (ACC) deaminase (A0U95_06530). ACC deaminase is suggested to be a key in the modulation of ethylene levels in plants by bacteria [26].

For secretion of extracellular proteins in the surrounding environment genes putatively encoding general secretory pathway proteins (Gsp) belonging to the type two secretion systems were found in L13-6-12 (e.g. A0U95_29195, A0U95_29200, A0U95_29205). Type six secretion systems have evolved in Gram-negative bacteria enabling them to interact with their host and to adapt to various microenvironments and specialized functions [27, 28]. Genes encoding components of the type six secretion system were found in L13-6-12 (e.g. A0U95_16935, A0U95_28720, A0U95_28755) putatively for interaction with eukaryotic organisms.

## Conclusions

In this report, we describe the complete genome sequence of *Pseudomonas brassicacearum* strain L13-6-12, a strain that was originally isolated from the rhizosphere of potato grown in Groß Lüsewitz, Germany and which was originally assigned as *P. fluorescens*. This strain was selected for sequencing based on its ability to protect plants from biotic stresses and to promote plant growth. It also has a collection of genes predicting volatile components and enzymes such as a protease, ACC deaminase and spermidine enabling L13-6-12 to protect and promote its host plant. Genes, encoding putative T2SS, T4SS and T6SS, allowing interactions with the host and the environment were detected, too. Further functional studies and comparative genomics with related isolates will provide insights into mechanisms useful for novel biotechnological processes for seed and root applications since the strain represent a promising candidate for improving of plant performance.

## Abbreviations

CDS: Coding DNA sequence
CLSM: Confocal laser scanning microscopy
COG: Clusters of Orthologous Groups
DAPG: 2,4-diacetylphloroglucinol
HCN: Hydrogen cyanide
HGAP: Hierarchical Genome Assembly Process
LB: Luria Bertani
NAII: Nutrient Broth II agar
NBII: Nutrient Broth II
RAST: Rapid annotations using subsystems technology
SMRT: Single molecule, real-time
SSM: Standard Succinate Medium
T2SS: Type 2 secretion system
